# Measurement of Frontal Midline Theta Oscillations using OPM-MEG

**DOI:** 10.1016/j.neuroimage.2023.120024

**Published:** 2023-03-12

**Authors:** Natalie Rhodes, Molly Rea, Elena Boto, Lukas Rier, Vishal Shah, Ryan M. Hill, James Osborne, Cody Doyle, Niall Holmes, Sebastian C. Coleman, Karen Mullinger, Richard Bowtell, Matthew J. Brookes

**Affiliations:** a Sir Peter Mansfield Imaging Centre, School of Physics and Astronomy, University of Nottingham, University Park, Nottingham, NG7 2RD, UK; b Cerca Magnetics Ltd. 2, Castlebridge Office Village, Kirtley Dr, Nottingham NG7 1LD; c QuSpin Inc. 331 South 104th Street, Suite 130, Louisville, Colorado, 80027, USA; d Centre for Human Brain Health, School of Psychology, University of Birmingham, Birmingham, B15 2TT, UK

**Keywords:** Magnetoencephalography, Optically pumped magnetometers, Working memory, Neural oscillations, Theta oscillations, Low frequency

## Abstract

Optically pumped magnetometers (OPMs) are an emerging lightweight and compact sensor that can measure magnetic fields generated by the human brain. OPMs enable construction of wearable magnetoencephalography (MEG) systems, which offer advantages over conventional instrumentation. However, when trying to measure signals at low frequency, higher levels of inherent sensor noise, magnetic interference and movement artefact introduce a significant challenge. Accurate characterisation of low frequency brain signals is important for neuroscientific, clinical, and paediatric MEG applications and consequently, demonstrating the viability of OPMs in this area is critical. Here, we undertake measurement of theta band (4–8 Hz) neural oscillations and contrast a newly developed 174 channel triaxial wearable OPM-MEG system with conventional (cryogenic-MEG) instrumentation. Our results show that visual steady state responses at 4 Hz, 6 Hz and 8 Hz can be recorded using OPM-MEG with a signal-to-noise ratio (SNR) that is not significantly different to conventional MEG. Moreover, we measure frontal midline theta oscillations during a 2-back working memory task, again demonstrating comparable SNR for both systems. We show that individual differences in both the amplitude and spatial signature of induced frontal-midline theta responses are maintained across systems. Finally, we show that our OPM-MEG results could not have been achieved without a triaxial sensor array, or the use of postprocessing techniques. Our results demonstrate the viability of OPMs for characterising theta oscillations and add weight to the argument that OPMs can replace cryogenic sensors as the fundamental building block of MEG systems.

## 1. Introduction

Magnetoencephalography (MEG) enables non-invasive assessment of human brain electrophysiology via the measurement of magnetic fields generated by neural current flow (Cohen, 1968). MEG is a powerful means to investigate brain function, providing millisecond temporal resolution and millimetre spatial precision. It is a well-established research tool (Baillet, 2017) and has important clinical roles, particularly in epilepsy (Rampp et al., 2019). However, the neuromagnetic field is extremely low amplitude (100 fT – 10 pT) and this makes engineering a MEG system challenging. Conventional MEG systems employ super-conducting quantum interference devices (SQUIDs), which can detect magnetic fields at the fT level (Hamalainen et al., 1993). However, most SQUIDs require cryogenic cooling to liquid helium temperature, and this brings about significant limitations: for example, to provide thermal protection to the participant, sensors must be held at some distance from the scalp, limiting sensitivity and spatial resolution. Additionally, the sensor array is fixed rigidly within a one-size-fits-all helmet which is typically built for adults, meaning data collection in people with smaller heads (e.g. infants) is challenging. Movement relative to the static sensors affects the accuracy of neuronal source reconstructions, and so subjects must remain still for long periods. Finally, the complex nature of the instrumentation makes MEG systems expensive to purchase and operate. For these reasons, the utility and uptake of MEG has been limited.

In recent years, MEG instrumentation has seen a fundamental change with the introduction of optically pumped magnetometers (OPMs). OPMs are small and lightweight magnetic field sensors that offer similar sensitivity to SQUID, but without the need for cryogenic cooling (for a review see (Brookes et al., 2022)). OPMs can be placed within a few millimetres of the scalp surface, decreasing source to sensor distance, thereby increasing the signal strength and spatial resolution (Boto et al., 2016; Boto et al., 2017; Iivanainen et al., 2017). Sensors can be mounted in a helmet and so move with the subject, meaning that – assuming background magnetic fields are controlled (Holmes et al., 2018; Holmes et al., 2019) – subjects can move during a scan (Boto et al., 2018). Helmets can adapt to fit any head size/shape meaning systems can have lifespan compliance (Boto et al., 2022; Hill et al., 2019). Also, because they do not require complex cryogenic infrastructure, OPM-based systems are simpler to site, maintain and operate than conventional instrumentation. MEG based on OPMs (OPM-MEG) therefore has exciting potential as a research and clinical tool, enabling new types of neuroscientific experimentation in naturally moving subjects, and in cohorts – particularly infants – who find conventional imaging systems hard to tolerate.

Despite the promise, OPM-MEG remains nascent technology and has some disadvantages over SQUID based instrumentation. One notable limitation is sensitivity to low frequency oscillations. Low frequency neural oscillations are extremely important neurophysiological markers: For example, in healthy adults, theta (4 – 8 Hz) oscillations are measurable from both the hippocampus (Barry et al., 2019; Tierney et al., 2021b) and the cortex (Jensen and Tesche, 2002; Onton et al., 2005), with the former related to tasks such as memory and navigation, and the latter associated with cognitive processing. In clinical research and practice, an excess of low frequency neural oscillations at rest in adults (also termed cortical slowing) is a well-established marker of disease with examples including Alzheimer’s (Jeong, 2004) and Parkinson’s disease (Stoffers et al., 2007). In infants and children, there is a general shift of oscillatory activity towards lower frequencies at younger age – for example, the alpha peak frequency is shifted to lower frequency in children compared to adults (Miskovic et al., 2015) and there is a trend of decreasing theta power, and increasing alpha power, with age (Hunt et al., 2019). These examples show the critical importance of high-fidelity low frequency measurement. Consequently, any viable OPM-MEG system must be capable of accurately characterising such activity.

The challenge in detecting low frequency activity with OPM-MEG arises due to three separate effects. Firstly, the inherent noise level of an OPM begins to increase at low frequencies (Boto et al., 2022). There are several reasons for this in the current generation of commercial OPMs, including the presence of magnetic impurities in the materials from which the sensor is made, and cross-talk with feedback loops for the vapour cell and laser temperature stabilization, which in turn lead to fluctuations in the sensor output. Second, all MEG installations are housed inside a magnetically shielded enclosure (MSE), typically constructed from multiple layers of high magnetic permeability and high electrical conductivity metal. The former reduces low frequency interference, and the latter attenuates high frequency magnetic fields. Such shielding techniques are well established; however the shielding of low frequency (<10 Hz) variations in magnetic field at the scale factors associated with MSEs designed for human measurements is challenging (Hill et al., 2022; Holmes et al., 2022), meaning higher interference at lower frequencies. Whilst this is also true for conventional MEG systems, SQUID-based sensors often measure magnetic field gradient, whereas OPMs measure the field itself, and the latter is influenced more by distant sources of interference. Finally, unlike SQUIDs, OPMs move with the head. Although this is a major advantage, if there is any remnant (temporally static) background field inside the MSE then sensors ‘see’ a changing magnetic field as they move. Since head movement is more likely to occur at low frequencies, this again disproportionately increases low frequency interference. In sum, the combined effect of inherent sensor noise, low frequency interference from the environment, and head movement make imaging low frequency effects with OPM-MEG a significant challenge.

Despite the difficulties, nascent evidence suggests that it should be possible to measure low frequency effects using wearable OPM-MEG. For example in a recent study, OPM measurements in unconstrained subjects reliably detected neural oscillations in the theta band, which localised to the hippocampus (Barry et al., 2019; Tierney et al., 2021b) during a task involving imagination of novel scenes. In addition, in a study on cortical tracking of speech in unconstrained subjects, the authors concluded that OPM-MEG is able to successfully measure brain activity at frequencies of 4 Hz and below (De Lange et al., 2021). However, at the time of writing there is yet to be a direct comparison of results from SQUID and OPM-MEG, when measuring low frequency neural oscillations. In this paper, we employ two tasks – a 4 – 8 Hz flashing visual stimulus, and an N-back (2-back) working memory task, both of which elicit neural effects in the theta band. Both tasks were carried out by the same 14 participants using SQUID and OPM-based MEG instrumentation and we make a quantitative comparison of the results. We test two hypotheses: first, if background magnetic field is controlled appropriately (Rea et al., 2021) and interference rejection methodologies used effectively, the SNR of OPM-based measures of theta activity will be comparable to that of SQUID-based metrics. Second, we hypothesised that individual differences in the electrophysiological signature of frontal midline theta oscillations would be preserved between the two scanners.

## 2. Materials and methods

### 2.1. Subjects and experimental paradigms

**Fig. 1.**
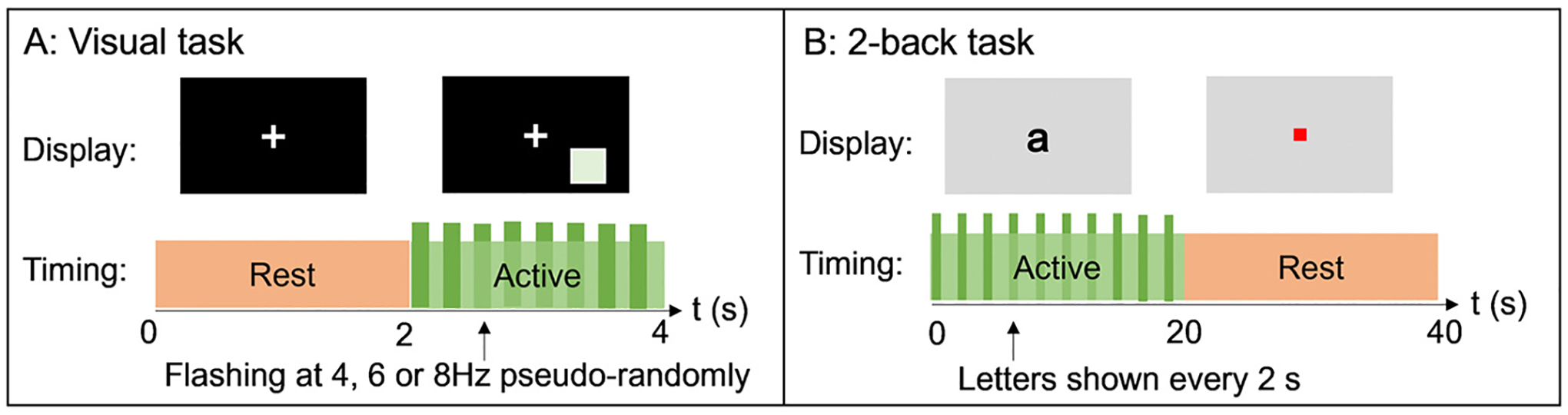
Paradigms – A) the visual task; B) the 2-back task.

Fourteen participants took part in the study (age range 27 ± 3; 8 identified as male, 6 as female). Each subject underwent two separate MEG recording sessions, with data collected by either OPM-MEG or SQUID-MEG. The order of these sessions was alternated (eight participants underwent OPM-MEG scans first; six participants were scanned first in the SQUID-MEG). The average time between sessions was 19 ± 10 days (mean ± standard deviation). In addition to MEG data, volumetric anatomical magnetic resonance images were acquired for each participant using a Philips Ingenia 3 T system running an MPRAGE sequence, with 1 mm isotropic spatial resolution. These anatomical images were used for subsequent coregistration between brain anatomy and the MEG sensor array geometries. All participants provided written informed consent prior to scanning, and the study was approved by the University of Nottingham’s Faculty of Medicine and Health Sciences Research Ethics Committee. A single scanning session comprised two experiments:

- **Visual task:** Participants passively viewed a visual stimulus – a green square on a black background, flashing at either 4 Hz, 6 Hz or 8 Hz. The stimulus was presented in the lower right quadrant of the participant’s field of view, subtending a visual angle of ~5° Stimulation was presented for 2 s, followed by 2 s rest period. This was repeated 120 times; with 40 trials for each frequency presented in pseudorandom order. The participant was asked to focus on a fixation cross at the centre of the screen throughout the recording, which lasted for 480 s. (See Fig. 1 A.)
- **2-back task:** A single trial comprised 20 s of letter presentations on a screen (vertical visual angle ~3°). One letter was shown every 2 s and was visible for 1 s (i.e. the gap between letters was 1 s). Participants were asked to press a button using their right index finger when the current letter matched that presented two letters previously (i.e., for a sequence of letters running ‘F’ ‘H’ ‘E’ ‘F’ ‘E’ ‘D’ subjects should press the button at the presentation of the second ‘E’). 20 s of ‘2-back’ was followed by 20 s of rest (Coleman et al., 2023), where participants were asked to focus on a central fixation dot on the screen. This was repeated for 25 trials, making the experimental duration 1000s. Similar 2-back tasks are well known to elicit frontal midline theta activity (Brookes et al., 2011). (See Fig. 1 B.)

Both paradigms were presented using back projection through a waveguide in the MSE onto a screen placed ~80 cm in front of the participant in the OPM-MEG system, and ~95 cm in the SQUID-MEG system. An Optoma HD39 Darbee projector with a refresh rate of 120 Hz was used to display the stimuli for both the OPM and SQUID recordings. A photodiode was attached to the screen at the location of the flashing square to monitor timings of the visual task. The timings of the letter presentations for the 2-back task were marked in the MEG data via triggers passed to the acquisition system using a parallel port. Subject responses to the 2-back paradigm were recorded using a fORP 4-button fibre optic response pad (Cambridge Research Systems Ltd.). This enabled measurement of correct/incorrect responses as well as reaction times.

### 2.2. OPM-MEG system details and data collection

#### 2.2.1. System overview

**Fig. 2.**
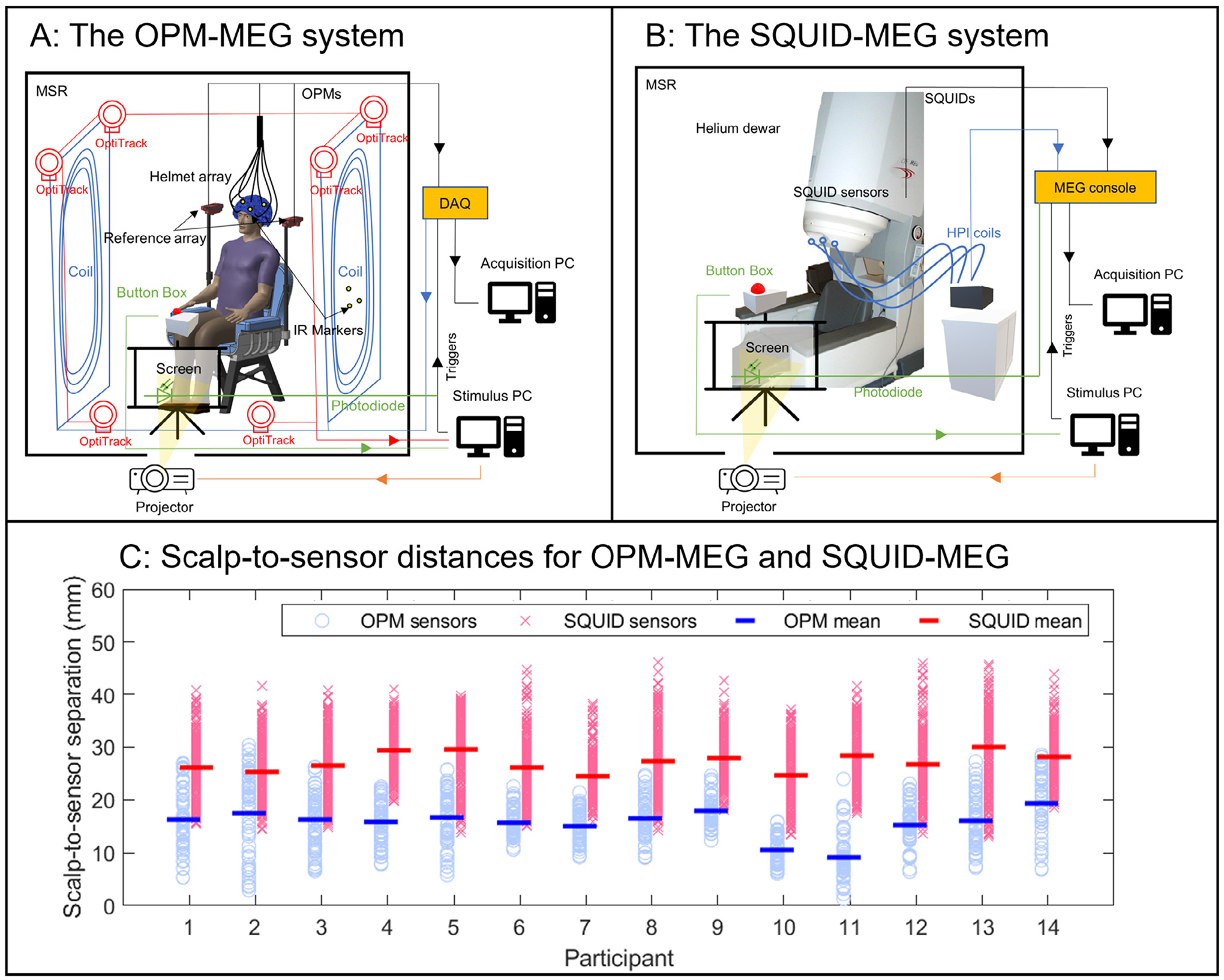
System overviews – A) OPM-MEG system; B) SQUID-MEG system; C) Distance from the scalp to the sensors for each participant, for both systems. Data points show values for all sensors; lines show the mean.

The OPM-MEG system (See Fig. 2 A) comprised 174 channels (58 triaxial OPM sensors (QuSpin Inc., Boulder, CO, USA)). The sensors were distributed uniformly across a rigid additively manufactured adult helmet (Cerca Magnetics, Nottingham, UK) providing whole-head coverage. The outputs of each OPM channel were fed into a National Instruments digital acquisition system (DAQ) and were sampled at 1200 Hz. A further 4 ‘reference’ OPMs (QuSpin Inc., Boulder, CO, USA) were placed immediately behind the subject to sample the background magnetic field. The system was housed in an OPM-optimised MSE (Cerca Magnetics Limited, Nottingham, UK) equipped with a bi-planar coil, which surrounded the subject and was capable of active field control (Cerca Magnetics Limited, Nottingham, UK). The room also housed a 6-camera motion tracking system (OptiTrack Flex 13, NaturalPoint Inc., Corvallis, Oregon, USA), which was used to monitor subject movement via infrared (IR) markers on the helmet and bi-planar coils. OPM control, data acquisition, and coil outputs were controlled using LabView (NI, Austin, Texas, USA) on a single ‘acquisition’ PC, whilst the stimuli and tracking cameras were controlled by a second ‘stimulus’ PC. ‘Triggers’ delineating the timing of events throughout the experimental paradigms were sent from the stimulus PC to the DAQ and were recorded alongside the MEG data.

#### 2.2.2. Precision magnetic field control

Rotation of an OPM through a static uniform magnetic field, or equivalently translation in a magnetic field gradient, results in a change in the measured magnetic field. At worst, these field changes can be so large that they cause non-linear changes in sensor gain, errors in sensitive orientation, and cross axis projection errors (CAPE), prohibiting accurate source reconstructions (Borna et al., 2022; Rea et al., 2021; Holmes et al., 2022). To avoid this, the background field over the array should be kept at < ±1 nT (Borna et al., 2022), ensuring OPMs provide reliable magnetic field measurements in the presence of movement. However, even at this low field, measured changes due to movement (whilst accurately sampled) can still be larger than the neuronal signals of interest. This is a particular problem at low frequencies, where movement often manifests. It is therefore essential that the background magnetic field be as low as possible if the subject’s head is to be unconstrained.

**Fig. 3.**
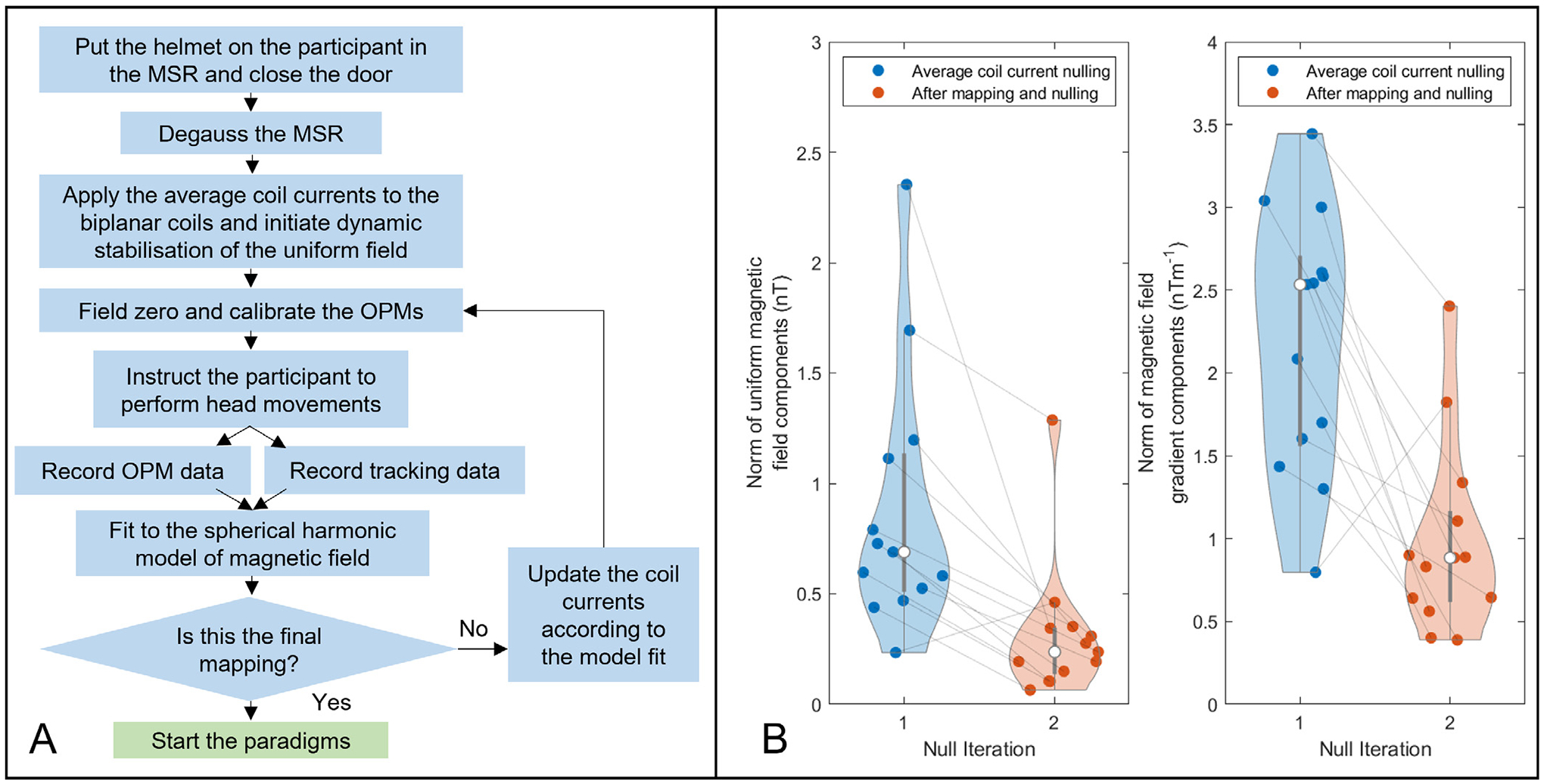
M agnetic field nulling results – A) Flowchart describing the field nulling process. B) The magnitude of the homogeneous magnetic field (left), and the linear field gradients (right) before and after application of the nulling procedure. Solid circles show individual data points and lines show the field trajectories for 13 subjects. The white circles show mean values across subjects.

Our system is housed inside an OPM-optimised MSE (Cerca Magnetics Limited, Nottingham, UK) equipped with a system for demagnetisation of the inner mu-metal layers. Demagnetisation typically leaves a magnetic field inside the MSE of ~ 3nT – too high for effective OPM operation with unconstrained subjects. To further reduce this field, active magnetic field control was employed using the bi-planar coils. The biplanar coil system is capable of generating the three uniform field components, and the five independent linear gradient fields, within a volume of ~ 0.4 × 0.4 × 0.4 m^3^ enclosing the participant’s head (Holmes et al., 2019). Nulling the background field in this volume thus allows head movements from a seated position. Here, the coil was used in two ways – firstly, our reference array of dual-axis OPM sensors (QuSpin Gen 1 sensors) was used to estimate the low frequency (< 3 Hz) temporal changes in the spatially uniform and gradient background magnetic fields (e.g. low frequency drifts caused by passing vehicles); such changes were fed back to the coil, which outputs an equal and opposite field to provide temporal stability (Holmes et al., 2019). Having stabilised the field in time, the remnant temporally static field was corrected via a nulling process first described by Rea et al. (Rea et al., 2021). Briefly, a series of head movements were performed by the participant and the translation and rotation of 5 triaxial OPMs in the helmet were captured using optical tracking. The measured field changes generated by movements of the same sensors were simultaneously recorded, combined with the movement data, and fitted to a spherical harmonic model to determine the static background field and field gradients present in the vicinity of the head. The coil was then used to offset these fields. The entire process can be repeated to improve performance. This technique has been shown to cancel remnant fields to < 300 pT. Here, unlike previous demonstrations of this technique (Rea et al., 2021; Holmes et al., 2022; Rea et al., 2022), rather than starting with the raw field following demagnetisation, an initial estimate of the background field (based on past studies) was used, and estimated currents were applied to the coils before the nulling procedure took place. Thus, the starting field was expected to be lower than the ~3 nT typically observed. This procedure (see also Fig. 3 A), which takes approximately 5 mins, was carried out for all participants.

#### 2.2.3. OPM-MEG data collection

The 3D printed helmet containing the OPMs was available in several sizes; prior to scanning, the subjects head circumference was measured, and the best-fitting helmet was selected and populated with OPMs. At the start of each scanning session, a 60 s “noise” recording was acquired with no subject present in the room, to assess levels of background interference and identify any problems with the system. Following this, subjects were seated comfortably on a patient support within the MSE, and the helmet was placed on their head. All subjects were completely free to move during the scan (though they were not specifically asked to do so). Once seated, the degaussing and remnant magnetic field nulling procedures were carried out. Following this, MEG data were recorded during the two paradigms. In addition, the experiment was also run once with no subject present, to obtain an empty room noise recording with equivalent duration to subject data.

To spatially coregister the MEG data to the anatomical MRI, two 3D digitisations of each participant’s head were acquired using an optical imaging system (Einscan H, SHINING 3D, Hangzhou, China). The first with the helmet on (to measure helmet position relative to the face) and the second with the helmet removed and a swimming cap used to flatten hair (to obtain an estimate of the whole head shape, including the scalp surface). A 3D surface representing the face and scalp was also extracted from the anatomical MRI scan. Coregistration was achieved via a 3-step process: 1) The two optical digitisations were segmented, leaving only points around the face, and were aligned to each other. 2) The second optical digitisation (with the helmet removed) was aligned to the surface extracted from the MRI. Combining steps 1 and 2 provided knowledge of the helmet position relative to the brain. 3) The locations and orientations of the OPMs relative to the helmet – known from the 3D printing process – were added, providing a complete coregistration of sensor position and orientation relative to the brain anatomy. This technique has been successfully used in previous work (Zetter et al., 2019; Hill et al., 2020). This final coregistration was subsequently used to enable forward modelling of the magnetic field generated by current dipoles in the brain.

### 2.3. SQUID system data collection

SQUID-MEG data were acquired using a 275-channel CTF system (CTF, Canada) in third-order synthetic gradiometer configuration, with a sampling frequency of 1200 Hz for the visual task, and 600 Hz for the working memory task (see also Fig. 2 B). The SQUID-MEG system is housed in a MSE (Vacuumschmelze, Hanau, Germany) to reduce the effects of external interference. As with all conventional MEG recordings, the subjects were asked to remain still throughout data acquisition. Prior to entering the scanner, three head position indicator (HPI) coils were placed on the subject’s head, and their locations inside the MEG helmet were tracked continuously throughout both experimental recordings. Motion greater than 5 mm was considered prohibitive, and data were discarded.

Following the experiment, a 3D digitiser (Polhemus, Colchester, Vermont, USA) was used to measure the location of the HPI coils relative to the head. The digitised surface was then fitted to the equivalent surface extracted from the anatomical MRI. This enabled coregistration of the MEG sensor geometry to brain anatomy. Note that this technique was selected (over the optical approach used for OPMs) as it represents a “standard” means to enable coregistration with conventional MEG.

Following coregistration the distance from all sensors to the closest point on the scalp surface was measured. This was carried out for both the SQUID and OPM systems and results are shown in Fig. 2 C. Notice that the OPM system gets sensors significantly closer to the head as expected.

### 2.4. Data analysis

All analyses were conducted in Matlab (MathWorks Inc.).

#### 2.4.1. Pre-processing

##### OPM-MEG:

OPM-MEG data were using a 4th order, two-pass Butterworth filter with a pass band of 1 – 40 Hz implemented in Nutmeg (Dalal et al., 2011). To remove bad trials, we first used an automated algorithm to remove any trials with noise variance greater than three times the standard deviation of the variance across all trials. Following this data were inspected visually and any channels or remaining trials displaying excessive noise were identified and removed. On average (across subjects) this resulted in the removal of 9 channels, and 4 trials. Homogenous field correction (HFC) (Tierney et al., 2021a) was then applied to remove interference that manifests as a spatially uniform field (Seymour et al., 2022).

##### SQUID-MEG:

A synthetic third-order gradiometry approach was used to reduce the effects of external magnetic interference in all SQUID-MEG data. Data were again filtered from 1 – 40 Hz using the same 4th order Butterworth bandpass filter. The same procedure to remove bad channels/trials resulted in removal of an average of 4 channels, and 1 trial.

#### 2.4.2. Source localisation

##### Visual task:

Data were further filtered using a narrowband 4th order Butterworth bandpass filter within a 4 Hz range around each of the stimulation frequencies (4, 6 and 8 Hz). The broadband (1 – 40 Hz) and narrowband filtered data were then segmented into trials. Source localisation was performed using a linearly constrained minimum variance (LCMV) beamformer (Robinson and Vrba, 1998). The brain was divided into 4 mm cubic voxels. Forward solutions were computed for every voxel using a single-shell head model based on individual MR data and a dipole approximation of neural current (Nolte, 2003). Covariance matrices were generated using the 1 – 40 Hz filtered data in a time window spanning the whole experiment (excluding bad trials); this was to ensure the maximum amount of data was used for covariance estimation (therefore minimising covariance matrix error (Brookes et al., 2008)). The covariance matrix was regularised using the Tikhonov method with a regularisation parameter equal to 2% of the maximum eigenvalue (Brookes et al., 2008). The optimised source orientation for each voxel was taken as that with the largest beamformer projected power (Sekihara et al., 2004). After generating the beamformer weights for each voxel, a pseudo-t-statistical approach was used to contrast narrowband source power between the “on” window (0 – 2 s relative to stimulus onset) and “off” window (2 – 4 s). The pseudo-T-statistic (T) was calculated as,

(1) T=wθTCONwθ−wθTCOFFwθ2(wθTCOFFwθ),

where ***w***_*θ*_ are the beamformer weights tuned to the location/orientation *θ*, and ***C***_*ON*_ and ***C***_*OFF*_ are the data covariance matrices computed during the “on” and “off” windows, respectively. Separate pseudo-T-statistical images were constructed independently for the 4 Hz, 6 Hz and 8 Hz stimuli. In each case we assessed the spatial signature of power changes at the fundamental frequency of stimulation. Voxels of interest in the visual cortex were selected based on the pseudo-T-statistical images, and a timecourse of the evolution of electrophysiological activity at these locations reconstructed (termed a ‘virtual electrode’ (VE)). The VE time course for each trial type was normalised (to give unit standard deviation) and averaged across trials. (The normalisation was to ensure that a single subject couldn’t dominate a group average). The Fourier Transform was then computed (for the “on”- and “off”-windows separately). Spectra were then averaged over individuals. Note that this procedure was applied identically for the OPM- and the SQUID-MEG data.

##### 2-back task:

Data were filtered to the theta band (4 – 8 Hz) using a 4th order Butterworth bandpass filter and segmented into trials. Source localisation was performed using the same beamformer and forward model described above. Covariance matrices were generated using theta-band data in a time window spanning the whole experiment (excluding bad trials) and were regularised again using the Tikhonov method with a regularisation parameter of 2% of the maximum eigen-value. A pseudo-t-statistical approach, as described in Eq. (1), was used to contrast theta band source power in the “on”- (5 – 20 s relative to trial onset) and “off” (24 – 39 s) contrast windows, producing volumetric images (again with 4 mm isotropic resolution) showing the spatial signature of the change in theta band power for each participant. The voxel in the frontal lobes with the peak pseudo-t-statistic for each participant was selected and a VE time course was extracted. VE data were processed in 2 ways: first, a Hilbert transform was computed, and the absolute value of the resulting analytic signal was used to generate the amplitude envelope (or “Hilbert envelope”) of theta activity. This was then averaged across trials; the baseline (computed as the mean of the envelope in the “off” window) was subtracted and the result divided by baseline amplitude to give a metric showing relative change in theta amplitude. The trial-averaged envelopes were then averaged across subjects. Second, the VE data were segmented into task (0 – 20 s relative to trial onset) and rest (20 – 40 s) windows, and a spectral amplitude estimate was derived using Welch’s overlapping segment averaging approach (with overlaps of 10 s). This method was applied to each trial separately, with results averaged over trials and participants. The empty room noise recording was also projected through the same beamformer weights that were derived for the 2-back data, and a spectral amplitude estimate was derived. This allowed for a direct comparison of spectral amplitude during task and rest, relative to sensor noise and background interference (excluding interference from the body (e.g. Magnetocardiogram) and movement artefact).

#### 2.4.3. Statistical analyses

The SNR was quantified for both tasks. In the visual task, SNR was defined as the amplitude of the spectral peak at the fundamental frequency (4 Hz, 6 Hz or 8 Hz) when the stimulus was on, divided by the average spectral amplitude in a 4 Hz window centred on the fundamental frequency when the stimulus was off. For the 2-back task, SNR was defined as the difference in the mean Hilbert amplitude between task performance (0 – 20 s relative to trial onset) and rest (20 – 40 s), divided by the standard deviation of the signal at rest. In both cases, SNR calculations were carried out at the individual and group level.

For the 2-back task, we expected significant inter-individual differences in both the amplitude and the spatial signature of the theta response (Brookes et al., 2010). Such differences would masque a traditional comparison of the two systems (involving group averaging) by increasing standard deviation across participants. However, we also expected that individual traits would be maintained between the two systems (i.e. a subject who had a high theta response in OPM-MEG would have a similarly high theta response in SQUID-MEG) (da Silva Castanheira et al., 2021). To test this, we carried out two separate analyses. First, we measured SNR at the individual subject level and plotted SNR in the SQUID system versus SNR in the OPM system. We expected this relationship to be dominated by individual differences, and therefore be approximately linear. Further we expected that the slope of a linear fit would tell us which system had the highest SNR (i.e. a slope of 1 would indicate parity between the systems). Second, we compared the spatial signatures of the pseudo-T-statistical images. Specifically, we measured spatial correlation (a Pearson correlation coefficient between the vectorised pseudo-T-statistical images) between all possible functional image pairs. With 14 subjects scanned in OPM-MEG and SQUID-MEG there are 14^2^ (= 196) possible combinations, of which 14 are within subject, and 182 between subject. We expected that the within-subject correlation values would be significantly higher than between-subject correlations. To test this statistically, we used a Monte Carlo method. Of the 196 correlation values, we randomly switched which 14 values were chosen as the within-subject correlations; doing this for 100,000 iterations enabled construction of an empirical null distribution and allowed us to estimate whether the real difference between within and between-subject correlations could have occurred by chance.

Finally, we were interested to see whether either the use of HFC (Tierney et al., 2021a) or the use of a triaxial array (Brookes et al., 2021) would affect the theta band signal-to-noise ratio. The above analysis was therefore repeated for 1) triaxial data with no HFC, 2) radial-only data with HFC and 3) radial-only data without HFC. Once again SNR was quantified (as above) and we explored the extent to which both array design (triaxial) and post processing, impacted the performance of our OPM system.

## 3. Results

### 3.1. Magnetic field nulling

Figure 3B shows the effect of magnetic field nulling. The plot on the left shows the magnitude of the spatially homogeneous components of the magnetic field inside the MSE. The plot on the right shows the magnitude of the linear field gradients. Both are shown before (blue) and after (orange) application of the nulling procedure. The individual markers show the fields/gradients for 13 of the subjects (individual subjects are connected by the grey lines) (unfortunately motion tracking data were unavailable for one subject). Importantly, in the case before nulling (“Null iteration 1 ”) initial voltages had been applied to the coil circuits to reduce the field in the room, based on measurements from previous studies (Rea et al., 2022). For this reason, the starting field magnitude (~ 0.9 nT) is lower than the typical ~3 nT which we would predict following degaussing. Nevertheless, optimisation via the nulling procedure reduced the field to an average of 0.31 ± 0.3 nT, highlighting the importance of this step in data acquisition.

### 3.2. Visual experiment

**Fig. 4.**
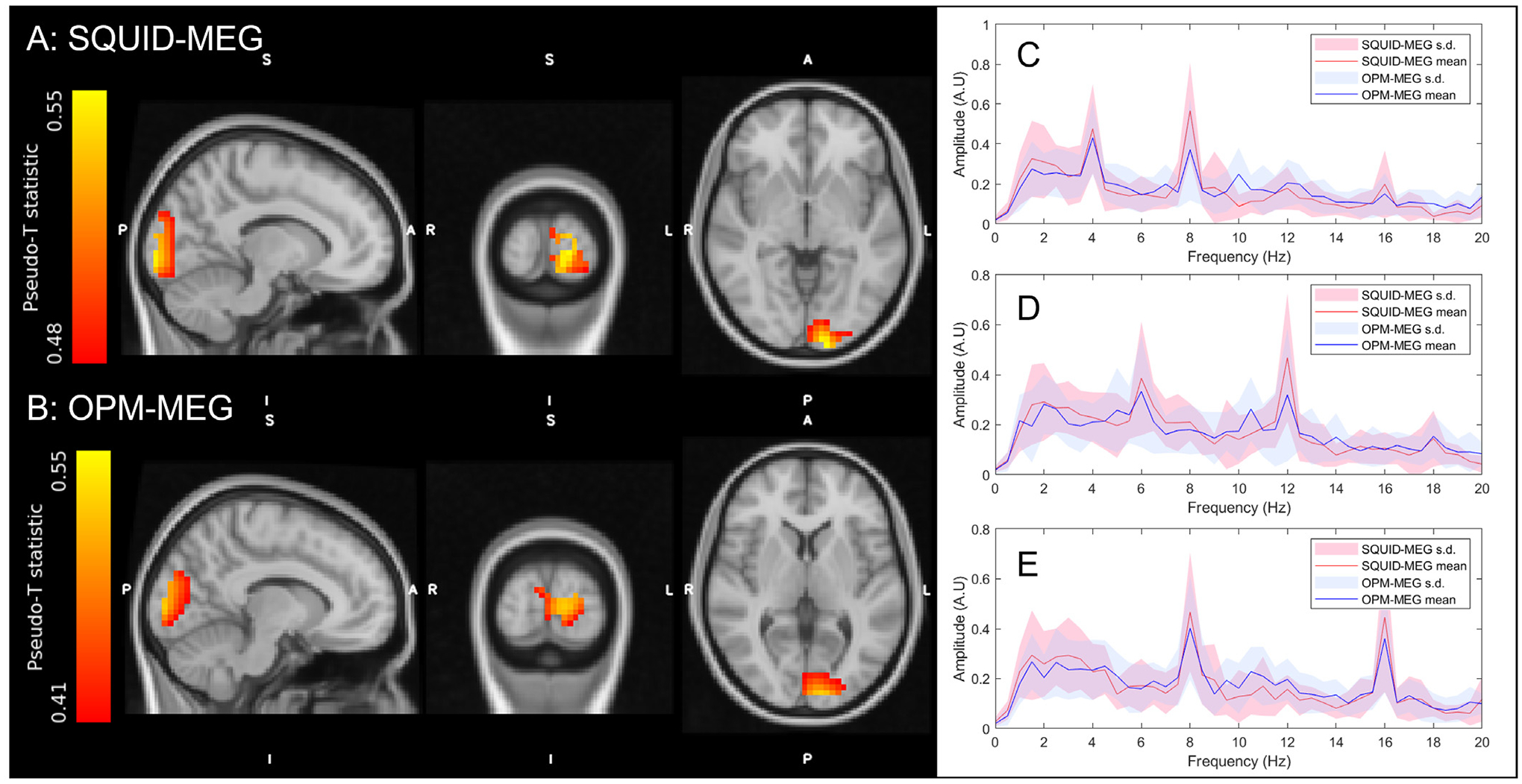
Visual experiment – A-B) Pseudo-T-statistical images showing the spatial signature of changes in oscillatory power at the fundamental frequency of stimulation (note that here 4 Hz, 6 Hz and 8 Hz have been combined). A) shows the case for SQUID MEG, B) shows the case for OPM-MEG. The locations of maximum theta change in A and B are separated by 12 mm. C-E) Subject averaged Fourier spectra of from the VE at the individual’s voxel with the peak T-statistic during stimulation. C) shows the case for 4 Hz stimulation; D) shows the case for 6 Hz stimulation; E) shows the case for 8 Hz stimulation. In all three cases, SQUID data are shown in red and OPM data in blue. Shaded areas show standard deviation across subjects.

Figure 4 shows the results of the visual experiment. Panels A and B show the spatial signatures of changes in oscillatory power. (All pseudo-T-statistical images have been spatially coregistered to the MNI brain (using FLIRT – (Jenkinson et al., 2002)) and averaged across subjects and frequencies). The result is similar for both systems, with the largest effect localising to the left primary visual cortex. This was expected given that the visual stimulus was presented in the lower right quadrant of the visual field, and maps retinotopically to the cortex. Panels C – E show power spectral data of VE’s extracted from the peaks in the pseudo-T-statistical images. In all cases, the data during stimulation are shown; panels C, D, and E, show 4 Hz, 6 Hz, and 8 Hz respectively. For all three stimulation types, peaks at the fundamental frequency and its harmonics are observed. Quantitatively, the peak oscillatory power changes for the two systems were separated spatially by 12 mm with the OPM-MEG appearing slightly anterior. The Pearson correlation coefficient between the two (vectorised) pseudo-T statistical images was 0.89. The SNR values for the OPM data were 2.0 ± 0.8, 1.7 ± 1.1 and 1.8 ± 1.1 for 4 Hz, 6, Hz and 8 Hz respectively. Equivalent values for the SQUID data were 2.3 ± 1.2, 1.8 ± 0.9 and 2.3 ± 1.1. The SNR differences between the two systems were not significant (*p <* 0.05) according to a Wilcoxon sum-rank test.

### 3.3. Working memory (2-Back) experiment

All 14 subjects performed well on the 2-back task. For the OPM data, subjects responded correctly to 95 ± 3% of the 2-back targets (mean ± standard deviation). The false positive rate (i.e. the percentage of non-target letters that were incorrectly identified as being a 2-back match) was 0.5 ± 0.5%. The average reaction time for correct responses was 0.576 ± 0.08 s. For the SQUID data, subjects responded correctly to 95 ± 5% of the targets. The false positive rate was 0.5 ± 0.5% and the reaction time for correct responses was 0.585 ± 0.10 s.

**Fig. 5.**
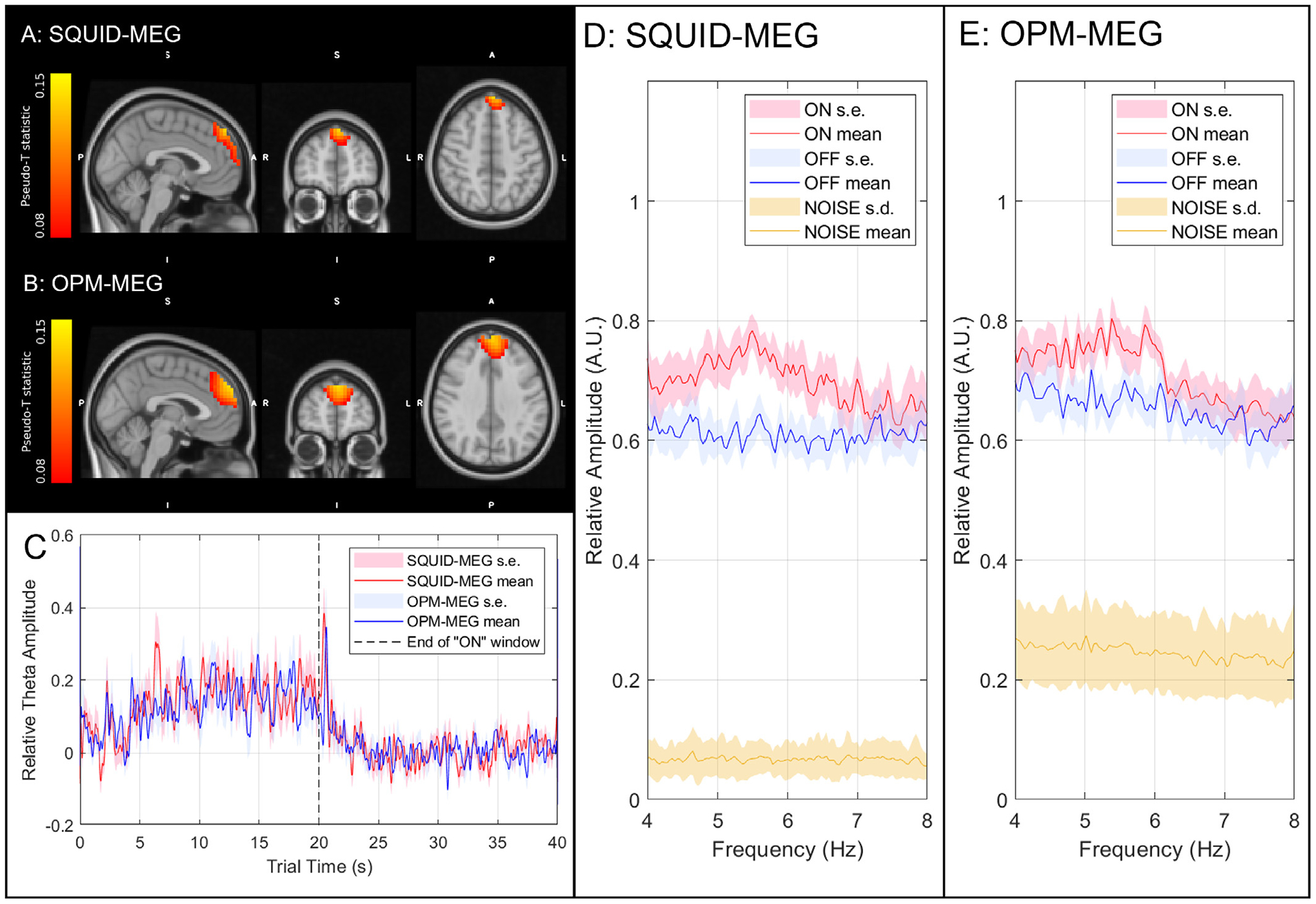
2-back experiment – A-B) Pseudo-T-statistical images showing the spatial signature of changes in theta band power (images show the average across subjects). A) shows the case for SQUID MEG, B) shows the case for OPM-MEG. The voxels with the highest theta change are separated by 13 mm between OPM and SQUID systems. C) Hilbert envelopes showing relative change in theta amplitude; the mean across subjects is shown and the shaded areas represent standard error. Red shows the SQUID recording, blue shows the OPMs. Stimulus cessation is shown by the dashed grey line. D–E) Subject averaged power spectral density from the virtual electrode during the task (red), rest (blue) and from the empty room noise recordings (yellow) for the SQUID measurements (D) and OPM measurements (E). The shaded areas for the task and rest windows show the standard error across the 14 participants. The shaded areas for the empty room noise recordings show standard deviation across the noise recording duration.

Figure 5A and B show the spatial signatures of the theta band response, averaged across subjects and overlaid on the MNI brain (using FLIRT – (Jenkinson et al., 2002)). In agreement with expectation, we observe a significant increase in the amplitude of theta oscillations that peaks in the frontal midline. Panel A shows the result for the SQUID measurement and panel B shows the case for OPMs. In both cases the result is spatially similar with the peak voxels in SQUID and OPM measurements separated by 13 mm. The Pearson correlation between the two (vectorised) images was 0.73. Fig. 5C shows timecourses of the Hilbert envelope, extracted from the peak locations in the frontal cortex (derived from the pseudo-T-statistical images) and averaged over trials and participants. A clear increase in theta amplitude is observed during the task (0–20 s) with strong similarity between MEG systems. The correlation between the SQUID and OPM envelope time courses was 0.76; the group level SNR of the SQUID and OPM systems was 5.14 and 5.02, respectively.

Figure 5D and E show the spectral amplitude of the SQUID- and OPM-derived virtual electrode data for the task period (red) rest period (blue). Empty room recordings are also shown (yellow). Note the clear elevation in theta amplitude during the task, relative to rest. As expected, the spectral amplitude of the empty room OPM recording increases with decreasing frequency; for example the theta band baseline was 1.22 times higher than the beta band baseline (16.5 fT/ √Hz for theta compared to 13.6 fT/ √Hz for beta when averaged over all sensors and noise realisations). This increase can be seen in the data in Fig. 5E. However, Fig. 5E also shows that the beamformer projected empty room spectral amplitude for the OPM system was around 4 times lower than the spectral amplitude of theta measurements in subjects at rest, suggesting that it is not the dominant noise source for theta measurement - this will be addressed further in our discussion.

**Fig. 6.**
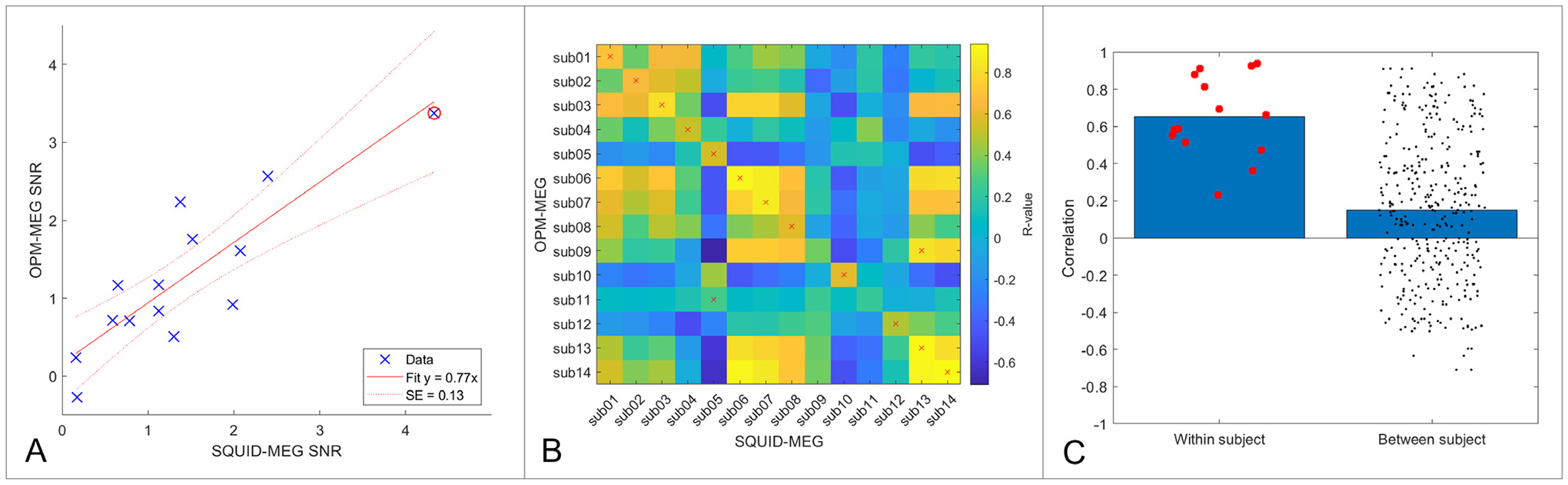
Individual differences – A) SNR of the theta band response measured in the OPM data, plotted against equivalent SNR measured for the SQUID data. Note the significant (*p* = 0.0001) linear relationship between the two systems. The red circled data highlights a single participant, whose strong theta response somewhat reduces the slope. B) Matrix of correlation values showing the spatial relationships between pseudo-T-statistical images of task induced theta change. Crosses indicate the highest correlation value within each row. Note crosses fall on the diagonal (i.e. within-subject correlation) for 12 out of 14 subjects). C) The same correlation values in (B) but plotted as within and between subject correlations. Dots show individual data points, and the bars represent the mean. The difference was significant (*p* = 0.00003) according to a Monte Carlo based statistical test.

Figure 6 shows results of our 2-back experiment at the individual subject level. Fig. 6A shows SNR for the SQUID system, plotted against SNR for the OPM system. Each data point represents an individual subject. Pearson correlation showed a significant (*r* = 0.86; *p* = 0.0001) relationship between the two measurements – i.e. those individuals who had a strong theta response in OPM-MEG data also tended to have a strong theta response in SQUID-based measurements. The slope of the line was 0.77 ± 0.13, indicating that the SNR of the OPMs was marginally lower than that of the SQUIDs. However, this was driven to a degree by a single subject since removing one data point (circled) changed the slope of the line to 0.85 ± 0.22, while still resulting in a significant relationship (*r* = 0.76; *p* = 0.003).

Figure 6B–C show the correlation values between pseudo-T-statistical images of change in theta power, for OPM-MEG and SQUID-MEG (i.e. each value shows how similar the two images are). In panel B, the matrix elements show all 196 possible correlation values between all subjects, the diagonal values represent within-subject correlation, whereas the off-diagonal elements represent between-subject values. Note subjects are ordered according to the SNR of their theta response (averaged across systems) (i.e. subject 1 has the highest SNR; subject 14 the lowest). These same quantities are shown in the bar chart in panel C, where within-subject correlations are on the left and between-subject correlations are on the right (dots represent individual values and the bars represent the mean). The data show that within-subject correlation (mean 0.65) is significantly higher than between-subject correlation (mean 0.15) (tested using our Monte Carlo method; *p* = 0.00003). In support of this, for 12 out of the 14 subjects scanned, when correlating their OPM-derived image with all 14 SQUID-derived images, the highest correlation (shown in Fig. 6 B by the red crosses) was with their own SQUID data (this analysis is sometimes termed neural fingerprinting (da Silva Castanheira et al., 2021) – with 12/14 subjects correctly identified). These results demonstrate that individual variation, measured via both SNR of the theta band response and its spatial signature across the brain, is maintained across two independent MEG recordings using very different instrumentation.

### 3.4. OPM-MEG artefact reduction

**Fig. 7.**
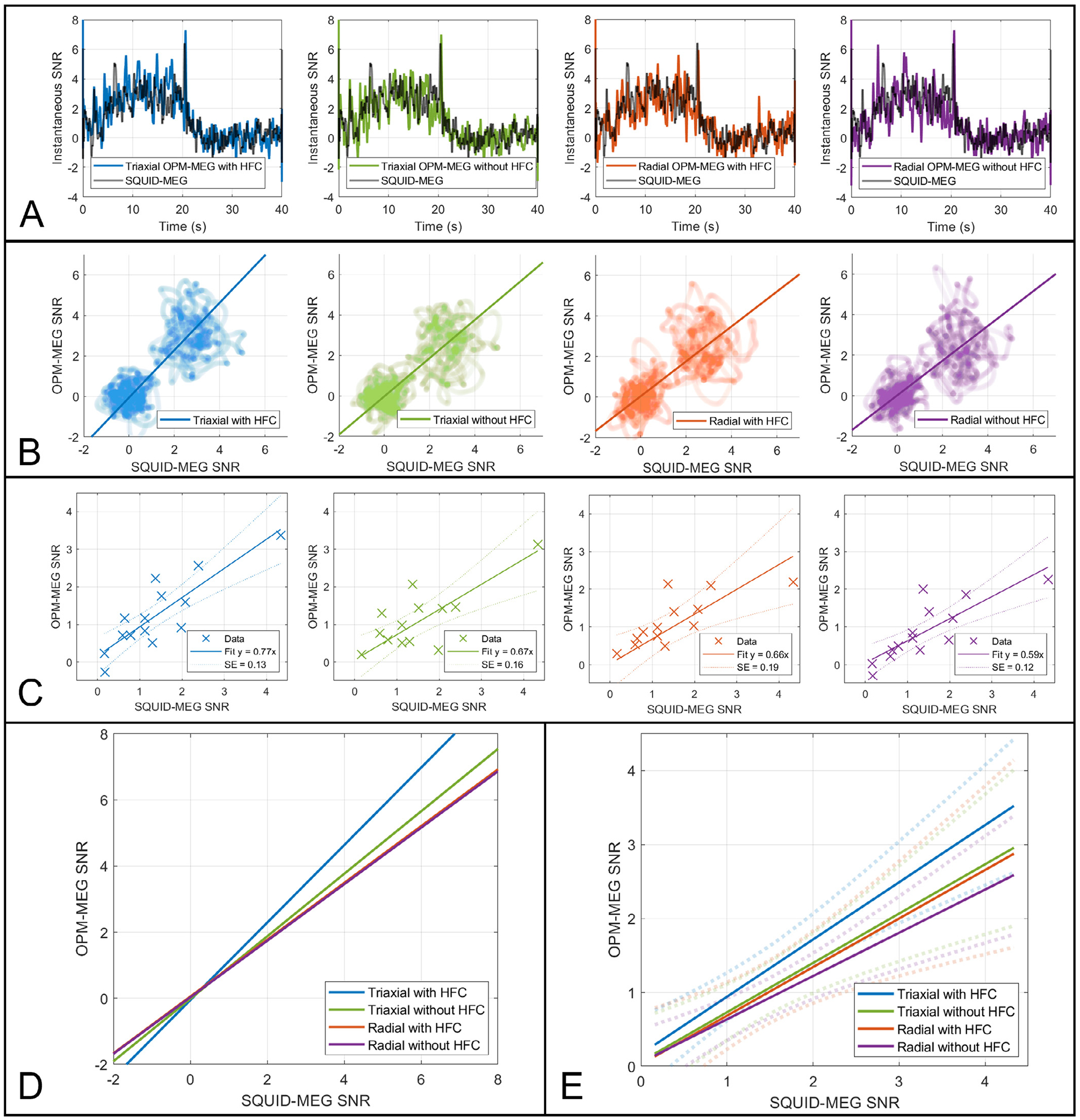
The effect of triaxial array design and homogeneous field correction – A) Subject averaged instantaneous SNR of both the OPM (coloured) and SQUID (black) theta band responses plotted against time. B) OPM derived plotted against SQUID derived instantaneous SNR. In both A and B, the 4 columns show triaxial data with HFC (far-left) triaxial data with no HFC (centre-left), radial data with HFC (centre-right) radial data with no HFC (far-right). C) SNR values from individual subjects; SQUID plotted against OPMs (i.e. equivalent to Fig. 6A). Order of columns as above. D) The line fits to the mean for the task and rest windows from the data in (B) overlaid. The slope of the line represents the relationship between SQUID and OPM instantaneous SNR values. E) the lines from C overlaid. Notice in both D and E that the slope of the line is diminished by removing tangential axes, and by removing HFC

Finally, we explored the extent to which the performance of OPM-MEG was influenced by both the use of a triaxial array (which is known to have good interference rejection properties (Brookes et al., 2021)) and homogeneous field correction. Fig. 7A shows four realisations of the theta band subject-average response, formulated as instantaneous SNR (i.e. instantaneous oscillatory amplitude divided by the standard deviation of the signal in the rest (20 s to 40 s) window) and plotted against time. The far-left hand panel shows triaxial data with HFC, the centre-left panel shows triaxial data without HFC, the centre right panel shows radial only data with HFC, and the far-right panel shows radial data without HFC. In all cases the coloured line shows OPM-MEG SNR, and the black line shows SQUID-MEG SNR (which is identical for all four plots and shown only for comparison). Fig. 7B shows the same data, but with the OPM SNR plotted against SQUID values. Here the slope of the line (which passes through the mean in the task and rest windows) is a representation of how OPM-SNR compares to SQUID SNR, at the group level. A slope of unity would indicate parity between the two systems. The four lines are overlaid in panel D. For triaxial data with HFC, the slope is 1.17, however, this drops to a value of 0.95 with no HFC; 0.86 for the radial-only recording (with HFC) and 0.85 when using radial recording and no HFC.

Figure 7C and E show a similar analysis, performed at the individual subject level. Fig. 7C shows individual subject SNR for OPM-MEG plotted against SQUID MEG. This is equivalent to Fig. 6A but here four realisations are shown – from left to right: triaxial data with HFC, triaxial data without HFC, radial data with HFC and radial data without HFC. Here the slope of the line indicates how OPM-MEG SNR compares to SQUID-MEG SNR at the individual level. The lines are overlaid in panel E. In agreement with the group average data, there is a marked effect of OPM-MEG performance, with a slope of 0.77 ± 0.13 for triaxial data with HFC, falling to 0.67 ± 0.16 with no HFC; to 0.66 ± 0.19 with radial only recording (with HFC) and to 0.59 ± 0.12 when using radial recording and no HFC. These effects show that both the use of triaxial sensing and homogeneous field correction influence the quality of OPM-MEG reconstructions.

## 4 Discussion

The measurement of low frequency neural oscillations is a significant challenge for wearable OPM-MEG due to increased noise/interference. Nevertheless, here we have shown that oscillations in the theta band can be measured reliably in unconstrained subjects using a 174 channel triaxial OPM-MEG system. We measured visual steady state evoked responses at 4 Hz, 6 Hz and 8 Hz, at a SNR that was not significantly different to a SQUID-based system (with 275 channels). Similarly, we measured task-induced increases in frontal midline theta oscillations during a 2-back working memory task, again with comparable SNR at the group level. Analysis of our working memory task at the individual level showed striking relationships between results acquired using the two different systems. Specifically, we showed a significant (linear) relationship between individual SNR values. Further, when measuring the consistency of the spatial signature of task-induced theta increases, within-subject correlation (0.65) was significantly higher than between-subject correlation (0.15) – meaning that both signal amplitude and spatial signature of theta change were maintained across the two independent recordings with very different scanner architecture. Finally, we showed that —whilst OPM-MEG could be used to characterise theta oscillations—SNR was dependant on both array design and HFC.

We had hypothesised that characterisation of low frequency activity would be difficult in wearable MEG for several reasons. Firstly, the internally generated noise inherent to the OPM sensor increases with decreasing frequency. Secondly, whilst environmental fields are shielded by the MSE, such shielding becomes more difficult as frequency decreases. Here, both the inherent sensor noise and low frequency environmental interference was assessed using our empty room recordings. Spectral analysis showed that, as expected, noise amplitude increases with decreasing frequency; however, Fig. 5E also showed that the beamformer projected empty room spectral amplitude was around 4 times lower when compared to the spectral amplitude of theta measurements in subjects at rest. It therefore follows that neither the low frequency interference nor the inherent sensor noise, both of which will be present in the empty room noise recording, is the dominant source of variance in the resting theta band signal. [As a side note, comparing Fig. 5D and E, we also point out that (as expected) the noise floor of a SQUID in the theta band remains lower than that of an OPM].

A third source of low frequency interference is movement. Any motion of the OPM array within a static background field manifests as a dynamic signal; if the background field is large this will contribute to the overall low frequency noise floor and could obfuscate neuromagnetic fields. Because movement is ‘slow’, such interference manifests at low frequency and hence we assumed it would be problematic for theta measurement. We attenuated movement artefacts at source by generating a background field as close to zero as possible. The background field was reduced to 0.31 ± 0.3 nT – a factor of ~10 better than we typically observe inside the MSE with no field nulling applied and ~160,000 times lower than the Earth’s static field. This demonstrates the critical importance of field nulling; without the use of bi-planar coils the movement artefacts would have been 10 times larger – a rotation of just 1° would generate a field shift of 52 pT (much larger than brain activity), and a 90° rotation would be sufficient to render our OPMs inoperable. Following our nulling procedure, even a full head rotation would maintain a total field shift of < 1 nT, meaning OPMs remain operational, and small rotations would generate artefacts of just a few pT. In the results presented (e.g. Fig. 5) we can’t isolate and quantify the effect of movement, and so its contribution to the overall noise floor is unknown. However, the similarity of the OPM and SQUID measurements shows that any movement artefact present in the data (following nulling) is not large enough to obfuscate signals from the neural sources.

There are also other sources of low frequency interference due to the presence of the participant, including magnetic fields from the heart, muscles, and eyes – all of which are larger than brain activity. In addition, fields from brain areas that are of no interest to the task (sometimes referred to as “brain noise”) will also generate theta band interference. It follows that these sources, in some combination, provide the dominant source of variance in the theta-band noise floor. This is likely true for both the SQUID and OPM measures and so explains the similarity in SNR across the two systems. For the OPM-recording, we used a combination of HFC and beamforming to reduce the effects of such fields; HFC reduces fields that are uniform across the sensor array; meaning mainly fields from distant sources (e.g. the heart) (Tierney et al., 2022). Beamforming is a spatial filtering technique that helps to remove fields that originate anywhere other than the brain region being probed. The performance of both techniques is known to be significantly enhanced by the use of a triaxial array (Brookes et al., 2021; Tierney et al., 2022). The utility of these techniques was shown in Fig. 7 , where SNR was improved by HFC. In addition, the use of triaxial (rather than conventional radial) sensors also improved SNR. To a degree, this latter finding is confounded because the comparison also involves changing the total sensor count. Nevertheless, our raw theta band measurements are clearly affected by residual fields, which originate outside the brain and are likely caused by a combination of movement and biological fields of no interest. Thus, for future studies of low frequency oscillations in OPM-MEG, post-processing to attenuate such fields should be considered essential.

Our primary result showed that theta band oscillations were well characterised by both OPM and SQUID-based MEG systems. Plots of OPM-MEG versus SQUID SNR showed an approximately linear relationship. This was expected due to the known variation in the theta response across individuals, which we expected to be maintained across scanners (because all our subjects were adults, there wasn’t a large variation in head size and consequently we didn’t expect this SNR relationship to be dominated by the inverse square law). Our results in Fig. 7 showed that OPMs demonstrated marginally higher SNR at the group level (a factor of 1.17) and marginally lower SNR in at the individual subject level (a factor of 0.77). Whilst potentially interesting, we stress that both of these metrics are susceptible to error and the subject group is relatively small.

Individual differences between subjects (both signal amplitude and spatial specificity) were consistently captured across both systems, despite acquisitions being (on average) 19 days apart. This is a small subject group, with just two repeat measures per participant and therefore this “neural fingerprinting” result should be treated with some caution. Nevertheless it provides some validation that our OPM-MEG system can indeed capture individual variance in a theta response. Variance between individuals is often treated as “noise” in neuroimaging studies, however these results add weight to an argument that between subject differences represent valuable sources of variance which should be properly modelled. More importantly, the strength of theta oscillations provides an important metric: frontal midline theta is induced by tasks with high cognitive demand and therefore a useful marker of healthy brain function; abnormally high theta oscillations can be a sign of cortical slowing – which itself denotes neurological problems – for example dementia (Prichep et al., 2006; Vecchio et al., 2014), and concussion (Lee and Huang, 2014); higher amplitude theta waves are observed in children and their reduction with age is a marker of neurodevelopment (Hunt et al., 2019). For this reason, the ability to accurately measure and characterise theta waves in individual subjects (and their variation across subjects) is an important property for any MEG system. Here we provide a benchmark showing that our 174-channel triaxial array design is, at minimum, capable of matching conventional SQUID-based instrumentation in characterising theta activity.

Finally, there are several limitations in the current study which should be addressed. We focussed on theta waves, with the lowest frequency at 4 Hz. This was for two reasons: first, the importance of theta waves in neuroscientific paediatric and clinical applications and second, the fact that theta oscillations are relatively easy to evoke in a repeatable and well-controlled manner. Nevertheless, oscillations below 4 Hz, e.g. delta waves, exist and are important biological markers. They are challenging to evoke in a controlled manner, being most prevalent during sleep, and in patients with e.g. concussion (Lee and Huang, 2014). Nevertheless, future studies should aim to generate similar OPM/SQUID comparisons for these lower frequencies, though this would likely involve a redesign of the OPM helmet to facilitate comfortable sleep.

There are also technical limitations of our current implementation of OPM-MEG: Perhaps the most obvious is channel count – here we contrast 174 OPM channels to 275 SQUID channels. Channel count is important both for signal acquisition (at the simplest level, the more channels you can average over, the better the SNR you will get) and for differentiating between signals from different brain regions. Our OPM-MEG system allowed triaxial measurement – this offers significant advantages in terms of differentiating brain signal from external interference, but also some disadvantages because the measured tangential components of the magnetic field are smaller in amplitude than the radial components. The result is that the total signal (summed over channels) will be higher for the 275 SQUID-based radial channels compared to the 174 triaxial OPM channels. With this in mind, the fact that we approximately achieve parity between systems is compelling. Nevertheless, extension to higher channel counts, where one can reap the advantages of triaxial sensing whilst also optimising overall signal amplitude is an extremely important step forward. An additional advantage of a triaxial sensor array is that it is robust to cross-talk (Boto et al., 2022), so sensors can be placed in close proximity without interference. There is therefore no fundamental limit on how close sensors can be, and future work should aim to construct higher density arrays which would improve spatial resolution and decrease brain noise. Aside from channel count, our field nulling techniques, whilst successful, still left a residual field of 310 pT and even in this low field, small rotations of the head could cause artefacts which are similar in magnitude to neural signals. At the time of writing, it is not clear what the limit on field nulling is – in principle it should be limited only by the extent to which we are able to prevent the field from changing during an experiment (around 0.13 nT (Holmes et al., 2019)), however small errors in head tracking, and potentially cable interactions between OPMs, are thought to affect the accuracy of modelling and therefore the extent to which we can null the background field. This said, there are no fundamental reasons why these techniques cannot be made more accurate, and thus the background field could be driven even lower. The noise level of the OPMs themselves remains higher than that of a SQUID. As pointed out above, it is unlikely that this represents the dominant form of noise for the measurements shown. Nevertheless further work to increase laser power and remove magnetic components from OPMs will be critical to driving the noise levels down closer to that of SQUIDs, which will help to improve SNR, particularly at low frequency. Finally, when modelling MEG data we require a forward model that provides an accurate reflection of magnetic fields at all of the MEG channels. Here we have used a single shell model (Nolte, 2003), which proved adequate. However, it is well known (Iivanainen et al., 2017) that the tangential components of magnetic field are more affected by volume currents than the radial components. It therefore follows that, as we move towards triaxial OPM systems, results could potentially be improved via the use of models – e.g. more complex boundary or finite element methods (Stenroos et al., 2007) that better account for volume currents.

## 5. Conclusion

The accurate measurement and characterisation of theta band neural oscillations is a critical requirement for any viable MEG system, with applications ranging from neurodevelopment to dementia. However, such characterisation poses a significant challenge in wearable OPM-MEG systems due to higher levels of inherent sensor noise, magnetic interference (which is harder to shield at low frequency) and movement artefact in a static background magnetic field. Here we have shown that our 174-channel triaxial system can capture theta oscillations with similar accuracy to that of SQUID-based MEG. Further, we showed that individual differences between participants – including the amplitude and spatial signature of induced frontal-midline theta responses – are maintained across systems. Our results therefore demonstrate the viability of a triaxial wearable OPM array to measure theta oscillations and add weight to the argument that OPMs can replace cryogenic sensors as the fundamental building block of MEG systems in the near future.

## Data Availability

Data will be made available on request.
